# Endoscopic vacuum-assisted closure combined with ventricular septal defect occluder for the treatment of complex anastomotic leak after esophageal cancer surgery: a case report

**DOI:** 10.1055/a-2749-2953

**Published:** 2025-12-08

**Authors:** Tao Chen, Haiping Peng, Qingyan Fu, Ke Yu, Xiang Ding

**Affiliations:** 1 658843 Department of Thoracic Surgery, Yueyang Central Hospital, Yueyang, China; 2 Department of Gastroenterology, Jishou University Affiliated Hospital Yueyang Central Hospital, Yueyang, China; 3 658843 Department of Gastroenterology, Yueyang Central Hospital, Yueyang, China

Anastomotic leak (AL) is a challenging complication of esophageal cancer gastroesophageal surgery, with an incidence of 7–20%, often leading to sepsis, organ failure or death. About 5–10% of AL patients develop secondary esophagotracheal fistula (ETF). Traditional surgical re-intervention has high mortality. We adopted a staged minimally invasive approach, endoscopic vacuum-assisted closure (EVAC), for AL closure, followed by the endoscopic placement of a ventricular septal defect occluder (VSDO) for secondary ETF. To the best of our knowledge, this is the first report of an endoscopic approach that combined EVAC and VSDO, offering a valuable paradigm for this complication.

A 63-year-old man was transferred to our hospital with esophageal cancer. Pathological examination confirmed high-grade squamous intraepithelial neoplasia and focal squamous cell carcinoma. He underwent radical esophagectomy via cervical–thoracic–abdominal triple incisions by our thoracic surgeons. On the 12th day after surgery, he had chest pain, with salivary frothy fluid in thoracic drainage. Gastroscopy showed 3 cm AL and 4 mm ETF ( Fig. 1 ). We prioritize EVAC for addressing fatal AL. A handmade device ( Fig. 2 ) was endoscopically inserted via forceps to the esophageal target site. The tube was connected to negative pressure for suction (−100 mmHg; Video 1 ). The device was changed every 3–7 days. When preparing for the sixth gastroscopy, gastroscopy showed that the AL had healed ( Fig. 3 ), totaling 25 days. Using the VSDO to close the ETF after 10 days, under bronchoscopy and endoscopy, a glide wire was passed through the esophagus into the trachea. An 80 cm 10 French sheath was advanced from the esophagus into the trachea through the fistula. A 13 mm VSDO was advanced over a sheath under direct visualization via a bronchoscope. Under direct visualization, the VSDO was successfully deployed at the ETF. Final assessment revealed that there was no residual flush and the discs were well seated ( Video 1 ). After resuming oral feeding, the patient was discharged. Half a month later, follow-up gastroscopy showed the occluder in place ( Fig. 4 ).

**Fig. 1:**
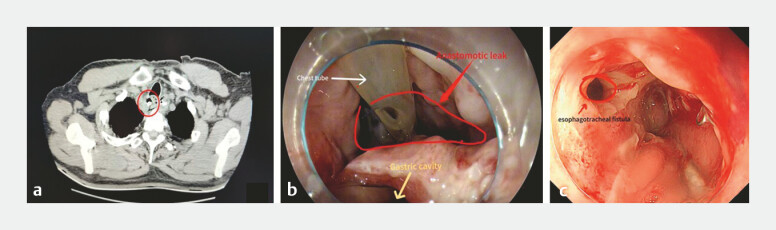
**a** CT revealed large anastomotic leak (location of the red circle) along with the right lower lung and right pleural effusions. **b** Gastroscopy showed 3 cm anastomotic leak (location of the red circle). **c** Gastroscopy showed 4 mm tracheoesophageal fistula (location of the red circle). CT, computer tomography.

**Fig. 2:**
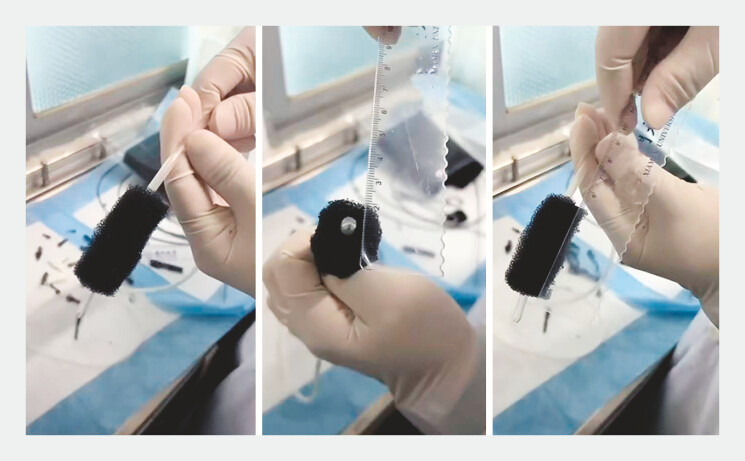
An appropriate EVAC kit was tailored based on the fistula size, including the nasogastric tube with side holes, sized black polyurethane sponge, secured by silk sutures. EVAC, endoscopic vacuum-assisted closure.

Dynamic CT demonstrates anastomotic leak and shows EVAC and VSDO detailed surgical procedure steps. CT, computer tomography; EVAC, endoscopic vacuum-assisted closure; VSDO, ventricular septal defect occluder.Video 1

**Fig. 3:**
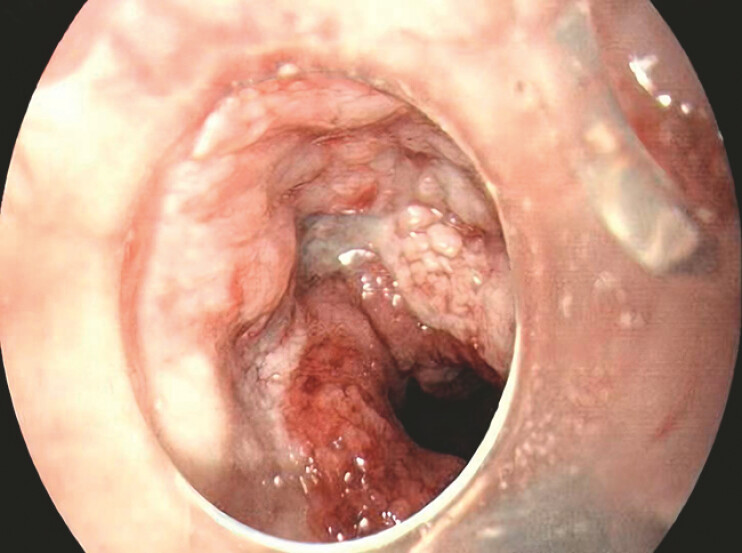
The sixth gastroscopy showed healing of the anastomotic leak.

**Fig. 4:**
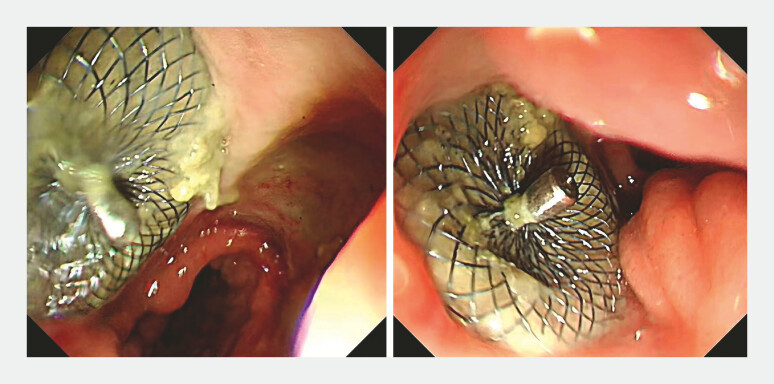
Follow-up gastroscopy showed that the anastomotic leak is closed, and the occluder is in place.

Endoscopy_UCTN_Code_TTT_1AO_2AO

## Notes

Endoscopy E-Videos https://eref.thieme.de/e-videos
*E-Videos* is an open access online section of the journal *Endoscopy* , reporting on interesting cases and new techniques in gastroenterological endoscopy. All papers include a high-quality video and are published with a Creative Commons CC-BY license. Endoscopy E-Videos qualify for HINARI discounts and waivers and eligibility is automatically checked during the submission process. We grant 100% waivers to articles whose corresponding authors are based in Group A countries and 50% waivers to those who are based in Group B countries as classified by Research4Life (see: https://www.research4life.org/access/eligibility/ ). This section has its own submission website at https://mc.manuscriptcentral.com/e-videos .
